# Accelerated Neuroimmune Dysfunction in Aged HIV-1-Infected Humanized Mice

**DOI:** 10.3390/ph17020149

**Published:** 2024-01-23

**Authors:** Chen Zhang, Hang Su, Emiko Waight, Larisa Y. Poluektova, Santhi Gorantla, Howard E. Gendelman, Prasanta K. Dash

**Affiliations:** Department of Pharmacology and Experimental Neuroscience, College of Medicine, University of Nebraska Medical Center, Omaha, NE 68198, USA

**Keywords:** HIV-1, aging, humanized mice, brain, transcriptomics, neuroimmune markers

## Abstract

Disordered immunity, aging, human immunodeficiency virus type one (HIV-1) infection, and responses to antiretroviral therapy are linked. However, how each factor is linked with the other(s) remains incompletely understood. It has been reported that accelerated aging, advanced HIV-1 infection, inflammation, and host genetic factors are associated with host cellular, mitochondrial, and metabolic alterations. However, the underlying mechanism remains elusive. With these questions in mind, we used chronically HIV-1-infected CD34-NSG humanized mice (hu-mice) to model older people living with HIV and uncover associations between HIV-1 infection and aging. Adult humanized mice were infected with HIV-1 at the age of 20 weeks and maintained for another 40 weeks before sacrifice. Animal brains were collected and subjected to transcriptomics, qPCR, and immunofluorescence assays to uncover immune disease-based biomarkers. CD4+ T cell decline was associated with viral level and age. Upregulated C1QA, CD163, and CXCL16 and downregulated LMNA and CLU were identified as age-associated genes tied to HIV-1 infection. Ingenuity pathway analysis affirmed links to innate immune activation, pyroptosis signaling, neuroinflammation, mitochondrial dysfunction, cellular senescence, and neuronal dysfunction. In summary, CD34-NSG humanized mice are identified as a valuable model for studying HIV-1-associated aging. Biomarkers of immune senescence and neuronal signaling are both age- and virus-associated. By exploring the underlying biological mechanisms that are linked to these biomarkers, interventions for next generation HIV-1-infected patients can be realized.

## 1. Introduction

Aging is characterized by a progressive physiological decline that leads to greater vulnerability to infectious, metabolic, degenerative, and cancerous comorbidities [1]. Aging involves a spectrum of molecular, cellular, and physiological changes that affect tissues and cellular functions [2]. The factors that control these functions remain poorly understood [3].

Age-related disorders have emerged as a critical research focus for people living with HIV (PLWH). In the United States, over half of the people living with HIV are over the age of 50, and the number is rising [4]. Aging is associated with frailty, cancer, diabetes, and cardiovascular and neurological diseases, which are also linked to advanced HIV-1 infection [5,6]. PLWH develop these conditions, even following antiretroviral therapy (ART) [7,8]. Viral loads, CD4+ T cell counts, infection duration, ART, substance-use-disorder-related toxicities, and opportunistic infections play important roles in disease acceleration [9].

The mechanism of the accelerated aging of the central nervous system (CNS) in PLWH remains undefined, in part, due to the lack of preclinical disease models. This has been overcome by the use of humanized mouse models [10], which can recapitulate HIV-1 infection and CNS-related abnormalities [11,12,13,14,15,16]. Our group and others have demonstrated that many of the viral infection-associated signatures and symptoms of CNS in PLWH can be reflected in such models; however, all prior rodent HIV studies were of short duration, lasting 4–5 months post-infection [12,13,14,17,18]. These data reflect prior longitudinal studies detected in infected persons [19,20,21,22].

In the current study, we aimed to bridge the gap between HIV infection and aging and find out how aging influences HIV-1-associated neurologic disease, using a suitable rodent animal model system. To achieve these goals, we infected hu-mice with HIV-1 at 5 months of age and obtained disease-linked biomarkers for 60 weeks. Viral infection and immune profiles were serially evaluated. This was done using transcriptomic analyses of brain samples from HIV-1-infected and age-matched uninfected hu-mice. This study was focused on human aging-associated markers. These were further validated through the immunofluorescence analysis of neuronal, glial, and oligodendrocyte markers. The results showed that the decline in CD4+ T cell was associated with levels of viral infection and age. Upregulations in C1QA, CD163, and CXCL16 and the downregulation of LMNA and CLU were identified as gene signatures. Ingenuity pathway analysis affirmed links to innate immune activation that included NK cell signaling, dendritic cell maturation, pyroptosis signaling, mitochondrial dysregulation, cellular senescence, and neuronal dysregulations, which was validated using qPCR and immunofluorescence tests.

## 2. Results

### 2.1. Viral and Immune Profile in Aged HIV-1-Infected Humanized Mice (Hu-Mice)

**Figure 1:**
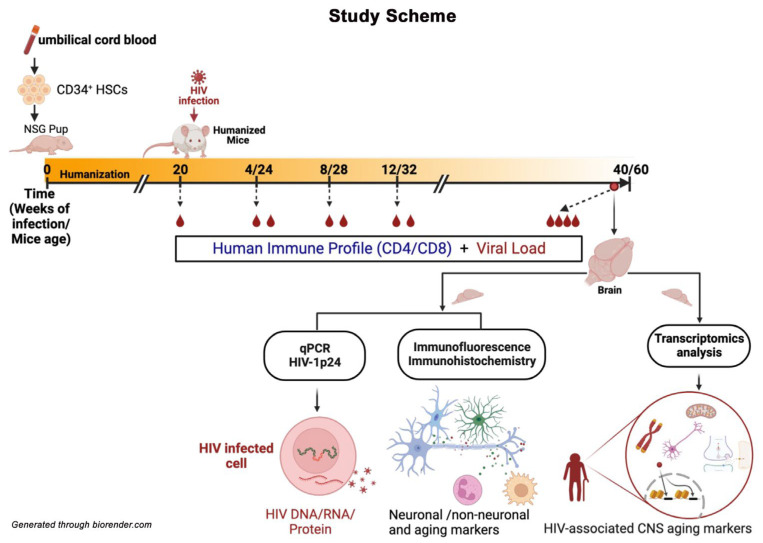
Study timeline. This includes the humanization of NSG mice after an intrahepatic injection of CD34^+^ hematopoietic stem cells, time after HIV-1 infection, and age. After the confirmation of humanization, mice were infected at 20 weeks of age with HIV-1_ADA_ intraperitoneally (IP) at a dosage of 1.5 × 10^4^ tissue culture infective dose 50 (TCID_50_) and maintained for another 40 weeks to observe aging-associated changes. The mice were bled, and human immune profile was measured at designated time points to look for human immune cells and viral presence. At the study end, mice were perfused with PBS, and the brains harvested for analyses.

The hu-mice were maintained up to 60 weeks of age and 40 weeks following HIV infection (Figure 1). Uninfected hu-mice at a similar age were used as controls. After the study, animal brains were collected for transcriptomic analysis for aging and virus-related biomarkers. To validate the findings, immunofluorescence staining, qPCR analyses, and quantifications were performed (Figure 1).

**Figure 2:**
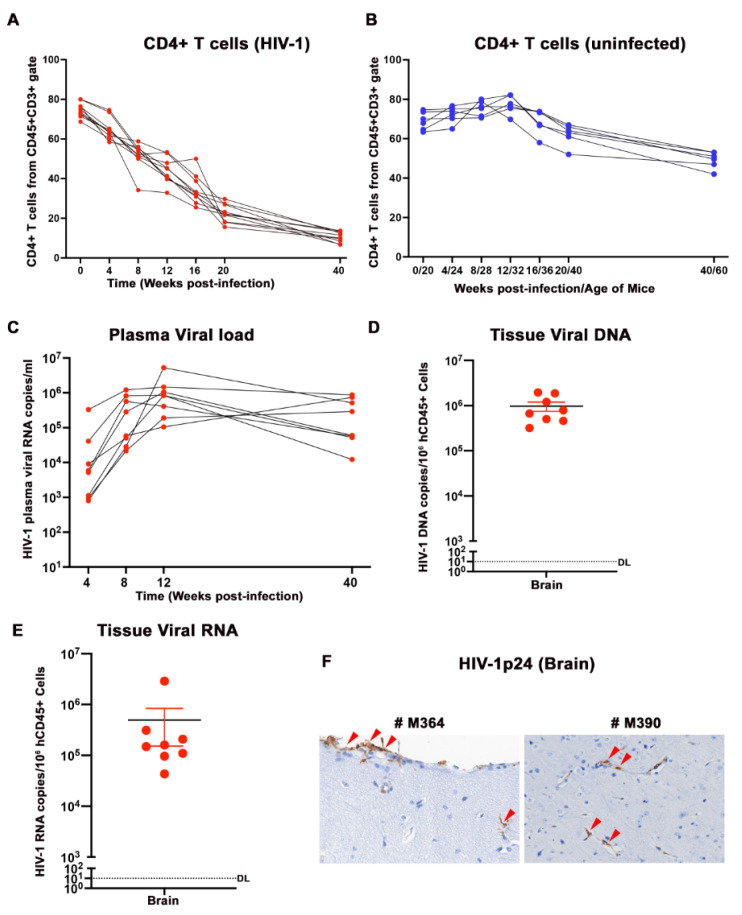
Immune and viral profiles of aged HIV-1-infected hu-mice. (**A**,**B**) Analyses of CD4+ T cell populations from the peripheral blood of HIV-1-infected (n = 8) and control (n = 6) mice for up to 40 weeks post infection. The mice were bled at designated time points, and human immune profile was measured for pan human CD45, CD3, CD4, CD8, CD19, and CD14 markers. A gradual decline in CD4+ T cells was observed in the aged and infected mice compared to the uninfected controls. (**C**) Dynamics of plasma viral load during progressive aging was measured once every 4 weeks. The detection limit (DL) of our assay was 200 copies/mL of viral RNA after dilution factor consideration. As the study was planned to look at the effect of aging, after 3 months of infection, hu-mice plasma was not tested until the endpoint of the study to increase the chance of survival of the animals. Tissue HIV-1 DNA (**D**) and RNA (**E**) from the brain of individual infected aged, humanized mice were evaluated at the study termination, using semi-nested real-time qPCR. The dotted line indicates the detection limit (DL) below 10 HIV-1 copies/10^6^ human CD45+ cells. A higher level of brain infection was observed in most of the infected and aged humanized mice brains. (**F**) Representative HIV-1p24 positive brain cells of infected hu-mice at 40× magnification. The pointed red arrows indicate the HIV-1p24 positive cells in the hu-mice brain.

CD4^+^ T cells were assayed in HIV-1_ADA_-infected (Figure 2A) and uninfected (Figure 2B) CD34-NSG hu-mice using flow cytometry from the total human CD45^+^ and CD3^+^ gates. A gradual decline in the CD4^+^ T cells was observed in HIV-1-infected mice from an average of 74.6% ± 3.4 before infection to 10.7% ± 3.53 at 40 weeks following infection. These results are reflective of advanced human infection. The uninfected control animals had a steady CD4^+^ T cell level during the study course, from 69.1% ± 4.9 at 20 weeks of age to 49.26% ± 6.84 at 60 weeks of age. Plasma viral load peaked around 8–12 weeks following infection, and all animals recorded VLs in the range of 8.45 × 10^5^ to 5.27 × 10^6^ RNA copies/mL. To allow the long-term survival of the hu-mice for study, the animals were not tested from 16 weeks until the endpoint of analysis. Sustained viral replication was observed at 40 weeks in all animals (1.22 × 10^4^ to 8.77 × 10^5^) (Figure 2C). To evaluate the level of viral infection in the brain and spleen at 40 weeks, real-time qPCR was performed targeting HIV-1-gag to quantify the tissue viral DNA (Figure 2D) and tissue viral RNA (Figure 2E). Robust viral infection was observed in the tissue samples analyzed for all infected animals, leading us to conclude that human cell types supporting viral infection can last for more than one year to study the long-term effects of HIV-latency and aging-associated pathways. Infected hu-mice brain sections were positive for HIV-1 p24 antigen, as analyzed using immunohistology (Figure 2F).

### 2.2. Aging-Linked Biomarkers in HIV-1-Infected Hu-Mice

To identify potential aging-associated biomarkers in the brain during chronic HIV infection, transcriptomic analysis was employed using the RT^2^ profiler Human aging PCR Array. The array was targeted to examine a broad range of gene expressions that are associated with genomic instability, telomere attrition, epigenetic alterations, inflammation response, cellular senescence, apoptosis, neurodegeneration, and synaptic transmission from both infected and uninfected aged hu-mice brain tissues.

**Figure 3:**
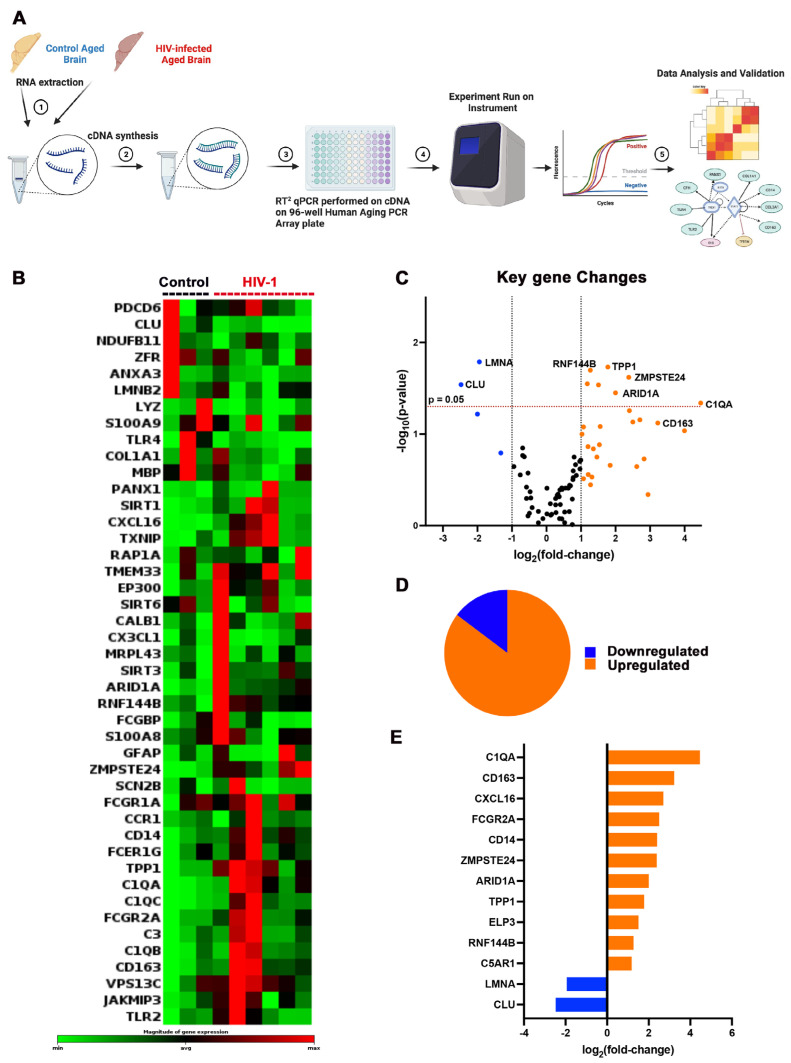
Transcriptomics of human age-associated genes from the brains of hu-mice. (**A**) Schematic representation of the RT^2^ profiler assay workflow utilized in this study. Total RNA was extracted from the brain tissues of aged, humanized mice infected with HIV-1 for about 10 months (n = 6) and uninfected control (n = 3) groups. RNA was reverse transcribed to cDNA. Subsequent amplification was conducted using the Human Aging PCR array for quantification and comprehensive analysis using RT^2^ Profiler PCR Array web-based data analysis software, version 3.5 (QIAGEN). (**B**) Heatmap showing differentially expressed genes. Red indicates highly expressed genes, and green indicates the genes with reduced expression in the aged, humanized mice brain samples. (**C**) Volcano map of differentially expressed genes. The orange and blues dots represent aging-associated genes that were upregulated and/or downregulated in the HIV-infected group as compared to the controls (|log_2_(fold change)| > 1). Each dot represents individual gene change. (**D**) Pie chart showing the proportion of upregulated (29) and downregulated genes (4) in aged, HIV-1 infected humanized mice brain samples. (**E**) Log2 fold change of the top-ranked differentially expressed genes in HIV-1-infected and aged mice compared to the age-matched control mice.

RNA from three control and six infected hu-mice brains was extracted, then converted to cDNA, followed by amplification using an RT^2^ profiler Human Aging PCR array targeting a total of 84 human aging-related genes (Figure 3A). Using Qiagen’s RT^2^ Profiler analysis software (Qiagen, Hilden, Germany) (https://geneglobe.qiagen.com/us/analyze), the expression of 44 human aging-associated genes was found significantly up/downregulated (*p* < 0.05) in HIV-1-infected as compared to uninfected animals, as shown in Figure 3B. The overall gene expression pattern was illustrated using a volcano plot in Figure 3C. HIV-1-infected hu-mice demonstrated an overall upregulation of the aging-associated genes analyzed as compared to age-matched controls. The key upregulated genes included RNF144B, TPP1, ZMPSTE24, ARID1A, C1QA, and CD163, while the major downregulated genes were LMNA and CLU. More differentially upregulated genes (29) were identified as compared to total downregulated genes (4), as shown in the pie chart (Figure 3D). The top 13 differentially expressed genes (either upregulated or downregulated), listed in Figure 3E, were ranked by the absolute value of their log2 (fold change). The overall upregulated pattern of aging-associated genes during chronic HIV infection in the humanized mice brains is in support of the clinical data with symptoms of accelerated aging in PLWH [23,24,25].

### 2.3. Pathway Enrichment Associated with Aged HIV-1-Infected Hu-Mice

**Figure 4:**
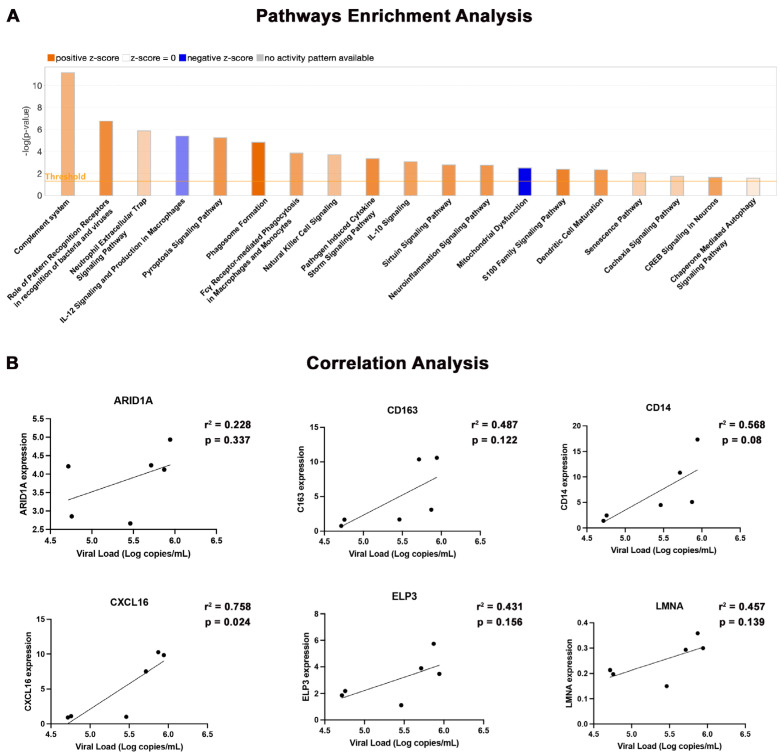
Canonical pathway analysis. (**A**) Gene network analysis was performed using IPA. Pathways are categorized based on the negative logarithm of the *p*-value, where *p*-value < 0.05 was considered significant. The color scheme of orange and blue indicates positive and negative z-scores, signifying elevated and reduced pathway activity, respectively. (**B**) Correlation of aging-related gene expression as assessed using RT^2^ profiler array from the brains of HIV-infected hu-mice.

To further investigate aging-associated pathways affected during chronic HIV infection, we utilized ingenuity pathway analysis (IPA) as described in the methods section and identified several over and under-expressed signaling pathways with multivariate biological functions. The top 19 altered signaling pathways are shown in Figure 4A.

Compared to uninfected controls, the major over-expressed signaling pathways in HIV-infected and aged mice brains were related to immune response, the complement system, and pattern recognition receptors. The other upregulations in signaling pathways were related to specific immune cell types and their functions, such as neutrophil extracellular trap signaling, dendritic cell (DC) maturation, and NK cell signaling, which are known to be responsible for the viral clearance and activation of adaptive immunity during HIV infection. We also observed the upregulation of pathways associated with phagosome formation and phagocytosis, which reflects the activation of macrophages and monocytes. The upregulation of pathways related to cell death, pyroptosis signaling, S100-family signaling, and chaperone-mediated autophagy pathways suggested the compensatory response to cellular disruption and cellular stress induced by HIV infection [26]. Cellular senescence pathways, usually associated with the aging process [27], were also found to be upregulated in the aged HIV-infected hu-mice.

Other than immune-response-related pathways, we found a significant activation of neuronal-signaling-related pathways, which included CREB and S100-family signaling. Interestingly, the neuroinflammation signaling pathway was found upregulated in our humanized mice brain samples, which is usually associated with HIV-related neurocognitive deficiencies.

Select pathways critical for immune response and cellular function were downregulated, including IL-12 signaling in macrophages. The other pivotal pathway was the mitochondrial function pathway, which oversees cellular energy production and is a hallmark of aging; this pathway also displayed diminished activity. Taken together, these results indicate a viral-induced dysregulation of neuroimmune and cellular functions of critical molecular pathways in HIV-infected aged animals.

Cellular gene expression was compared against plasma viral loads at the study’s endpoint (Figure 4B). We did not observe a statistically significant correlation for LMNA, CD14, ELP3, CD163, and ARID1A genes, except CXCL16, but a trend was found wherein samples with a higher viral load consistently exhibited an elevated expression of aging-associated genes.

### 2.4. Multispectral Immunofluorescence Imaging

**Figure 5:**
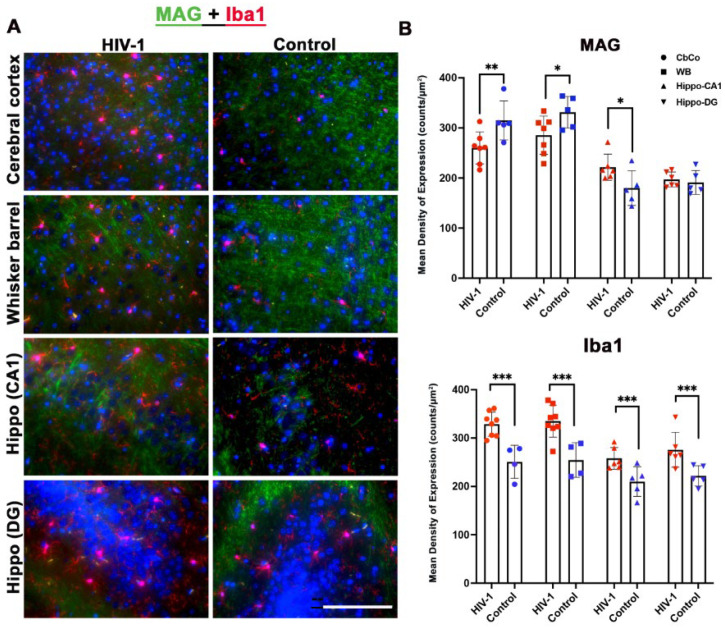
Immunofluorescence MAG and Iba-1 staining. (**A**) Representative images from the cerebral cortex (CbCo), somatosensory cortex or whisker barrel (WB), hippocampal cornu ammonis (Hippo-CA1), and dentate gyrus (Hippo-DG) at 40× magnification. MAG is in green, Iba1 in red, and nuclei stained with DAPI in blue. (**B**) Quantification of MAG- and Iba1-positive expressing cells in both HIV-infected and control mice as assessed using nuance multispectral imaging. Data are derived from a mean of n = 8 images per brain region and presented as mean ± SEM. Statistically significance differences between the HIV-1 and control and aged groups are shown as follows ***, *p* ≤ 0.001; **, *p* ≤ 0.01; *, *p* < 0.05.

We then validated our transcriptomics data obtained from the HIV-infected and aged animals with end-organ expression of markers specific for neurons, glial cells, oligodendrocytes, and other aging-specific cell types, using multispectral immune-fluorescence imaging on multiple regions of brain sections from the same animals. We quantified replicate brain areas in both HIV-1-infected and age-matched control groups. The representative immunofluorescence staining of the cerebral cortex (CbCo), somatosensory or whisker barrel (WB), hippocampal cornu ammonis (Hippo-CA1), and hippocampal dentate gyrus (Hippo-DG) regions revealed distinct expression patterns for oligodendrocytes (MAG) and microglia (Iba-1) markers, as depicted in Figure 5A.

Quantitative analyses based on multispectral imaging further revealed statistically significant differences in the expression levels of both MAG and Iba-1 antigens in HIV-1-infected and aged animals as compared to uninfected mice. For the oligodendrocyte marker MAG, a significantly reduced expression was observed in HIV-infected and aged mice compared to the uninfected group in the CbCo, WB, and hippo-Ca1 regions. For the microglial activation marker Iba-1, there was a significantly higher expression in all the brain regions analyzed in the HIV-1-infected group as compared to control animals (Figure 5B).

**Figure 6:**
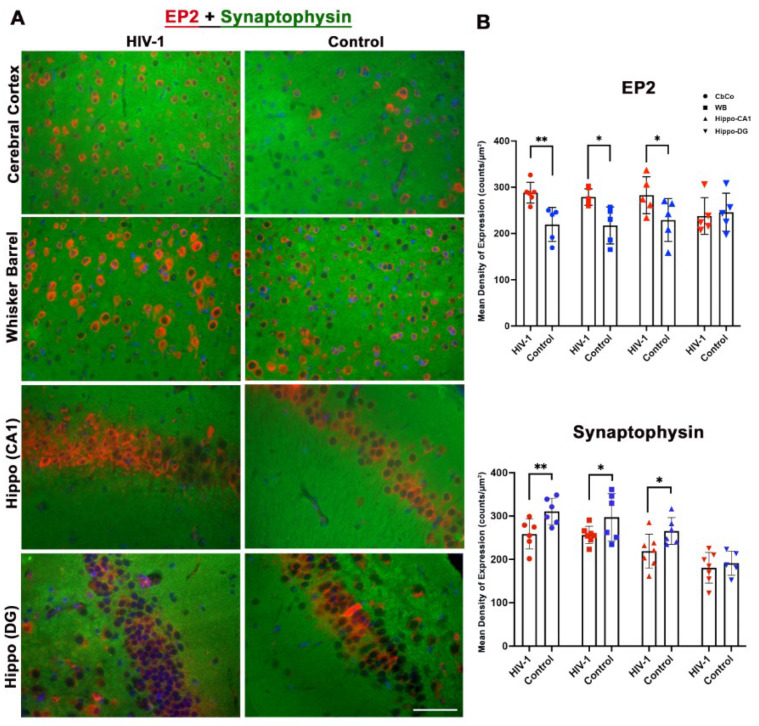
Immunofluorescence staining and quantification of EP2 and synaptophysin. (**A**) Representative images from CbCo, whisker barrel, hippocampal Hippo-CA1, and Hippo-DG at 40× magnification. Staining is shown for the nucleus (DAPI: blue), EP2 (red), and synaptophysin (green). (**B**) Quantification of EP2- and SYN-positive expressing cells in both HIV-infected and control mice as assessed using nuance multispectral imaging. Data are derived from a mean of n = 8 images per brain region and presented as mean ± SEM. Statistically significance differences between the HIV-1 and control and aged groups are shown as follows **, *p* ≤ 0.01; *, *p* < 0.05.

We then looked for the expression of an aging-specific marker, prostaglandin-2 receptor (EP2), and the neuronal integrity marker synaptophysin by dual staining the brain tissues with EP2 and SYN antibodies (Figure 6A). Quantitative analyses indicated a significant increase in EP2 expression in the CbCo, WB, and Hippo-CA1 regions of infected aged mice as compared to uninfected hu-mice brains. A significant decrease in synaptophysin expression in the CbCo, WB, and Hippo-CA1 regions of infected aged mice was observed (Figure 6B).

**Figure 7:**
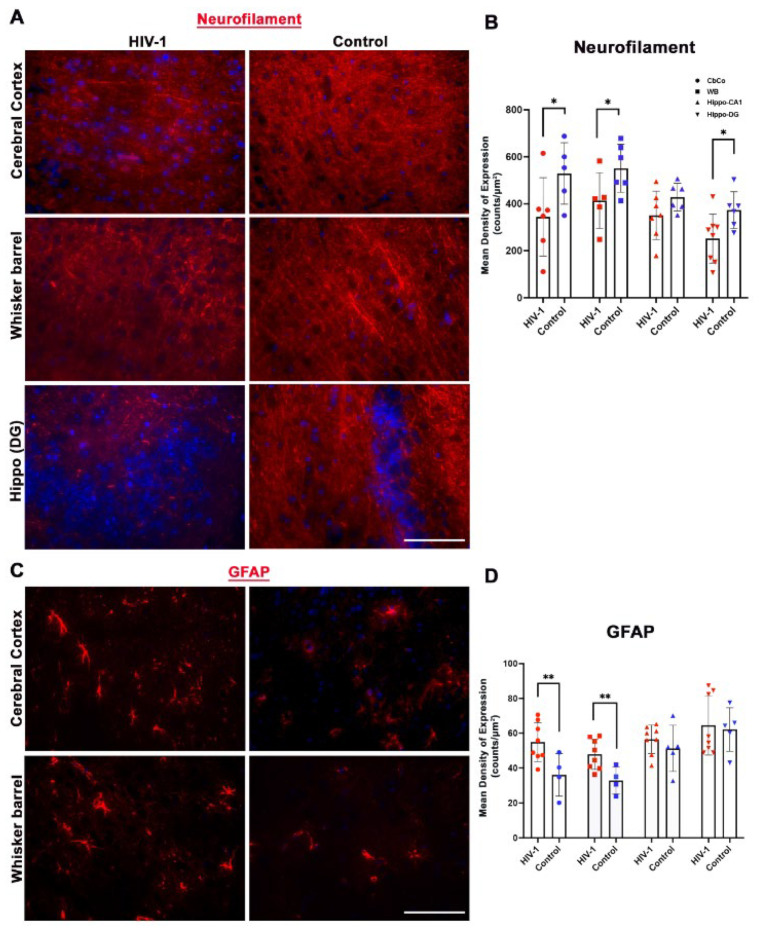
Immunofluorescence staining and quantification of neurofilament and astrocytes. (**A**) Representative images from CbCo, whisker barrel, and Hippo-DG at 40× magnification. (**B**) Quantification of NF-positive expressing cells in both HIV-infected and control mice as assessed using nuance multispectral imaging. Staining is shown for the nucleus (DAPI: blue) and NF (red). (**C**) Representative images from CbCo and whisker barrel at 40× magnification. (**D**) Quantification of GFAP-positive expressing cells in both HIV-infected and control mice as assessed using nuance multispectral imaging. Staining is shown for the nucleus (DAPI: blue) and GFAP (red). Data are derived from a mean of n = 8 images per brain region and presented as mean ± SEM. Statistically significance differences between the HIV-1 and control and aged groups are shown as follows **, *p* ≤ 0.01; *, *p* < 0.05.

A notable downregulation of the neurofilament-specific marker was observed in HIV-infected and aged hu-mice in the CbCo, WB, and Hippo-DG subregions of the brain, as shown in Figure 7A,B. Additionally, the astrocytic-GFAP expression was found to be upregulated in the CbCo and WB regions of the brain in aged and HIV-1-infected animals (Figure 7C,D). A trend towards significance was observed with increased GFAP-specific expression in the hippocampus.

## 3. Discussion

PLWH often exhibit complications of premature aging such as cardiovascular [28], cancer [29], renal [30], metabolic [31], and neurocognitive disorders [32,33], even when virus infection is ART-suppressed. The mechanistic reasons could be due to a combination of factors that include persistent immune activation and inflammation, genomic and epigenetic alterations, cell aging, mitochondrial energy [34,35,36], and metabolic shifts [31,37], with or without ART [38]. Several mechanisms are linked to PLWH premature aging [27,39,40,41,42]. This primarily occurs by increasing oxidative stress [43], leading to premature aging in PLWH. Therefore, it is imperative to identify factors linked to accelerated aging as these factors could affect the quality of life.

The use of CD34-NSG hu-mice containing a functional human immune system enables chronic HIV infection and allows for studies of accelerated aging. Prior studies in these models observed the relationships between peripheral blood markers and behavior [44]. Herein, CNS-specific aging markers were investigated. NSG hu-mice were previously used to study HIV-associated peripheral immune and CNS biomarkers in up to 38–40 weeks of hu-mice age, reflecting 18–20 weeks of HIV-infection [12,13,14]. Hu-mice in the current study were used to uncover relationships between aging and persistent viral infection at 60 weeks. We also asked how long a functional human immune system could sustain viral infection in hu-mice to study aging. Sustained viral replication was observed in the plasma until 40 weeks post infection (Figure 2C), which was equivalent to 60 weeks following human cell reconstitution. This was confirmed in tissues (both brain and spleen) as measured through viral DNA and RNA quantifications using real-time qPCR (Figure 2D–F). A progressive decline of human immune cells, particularly CD4+ T lymphocytes, during HIV-1 infection (Figure 2A,B) was observed in infected animals reflecting the natural disease progression.

Current evidence of aging-associated biomarker in PLWH includes monocyte activation markers [45], neurofilament light chain (NFL) [9,45], inflammatory cytokines, and cell-based activation [46]. However, these markers are from peripheral blood, which may not fully reflect the neuropathology associated with aging. A few studies utilizing cerebrospinal fluid (CSF) samples from ART-treated patients have revealed an accelerated aging profile related to neuronal injury, neurotransmitter dysregulation, altered glial responses, and the brain waste disposal system [47,48,49]. However, these studies are restricted by the small sample size of the CSF-based studies and their internal variability, limiting their applicability to a broader HIV-infected and ART-treated population.

While clinical evidence predominantly comes from the peripheral immune system, our study evaluated age-associated transcriptomic changes in the brain of CD34-hu-mice during chronic HIV-infection. We observed a significantly elevated innate immune response in aged, infected hu-mice as compared to age-matched controls, which includes the upregulation in complement system activation, proinflammatory chemokines, and all the pivotal sub-cellular components of innate immunity (macrophages, DCs, and neutrophils), indicating a robust activation of immune response in the hu-mice brain with aging. We observed upregulation in CXCL16 and an overactivation of the complement system in aged, infected hu-mice, which may contribute to chronic inflammation and has been shown to be associated with accelerated aging [39]. Monocyte activation markers, CD163 and CD14, were also upregulated in infected and aged hu-mice. These data corroborated previous findings from both peripheral blood and CSF in patients with age-associated comorbidities [45,50,51,52,53]. The C1QA gene, a critical component of the classical complement pathway, exhibited a marked upregulation in the infected hu-mice brains, indicating an activation of innate immune response within the CNS. TPP1, a nucleoprotein pivotal for maintaining telomere integrity and homeostasis, showed elevated levels in our aged, infected samples. However, TPP1’s function in chronically HIV-infected patients remains unclear [54,55]. We noted an upregulation of the genomic instability marker, zinc metalloproteinase ZMPSTE24, which has been associated with increased levels of inflammation and an increase in progerin mRNA, both recognized as indicators of aging [56]. Additionally, other aging-associated genes, including epigenetic regulation marker (ARID1A) and proteostasis regulation marker RNF144B, were also found to be upregulated.

Other than the upregulated genes discussed above, Lamin A/C (LMNA) and Clusterin (CLU), which were involved in metabolic laminopathies and apoptosis, were found to be significantly downregulated in our HIV-infected and aged group [57,58,59].

The major over-expressed signaling pathways in HIV-infected hu-mice brains, as compared to uninfected aged controls, were related to immune responses supporting previous clinical findings [60,61]. This includes neutrophil extracellular trap signaling, DC maturation, and NK cell signaling, which are all responsible for the viral clearance and activation of adaptive immunity during HIV infection [60,62,63]. The other affected pathway of importance is diminished activity of mitochondrial function, which could be associated with potential cellular energy deficits in PLWH [38]. We also observed aging-related alternations such as cellular senescence, a loss of proteostasis, and epigenetic alterations in our infected and aged hu-mice, suggesting that chronic CNS viral infection may be contributing to premature aging through various mechanisms beyond immune activation and neuroinflammation. Despite the differences between the murine and human brains, our study is the first to find predominant alterations in pyroptosis and subtle changes in cellular senescence and autophagy pathways, which could be playing an indirect role in accelerated aging during chronic HIV-infection. Additionally, we found an elevation of neuroinflammation and neuronal dysregulation pathways (the CREB and S100 Family signaling) in the infected aged samples, reflecting the interplay between the immune system and the CNS in hu-mice.

Immunofluorescence analyses evaluated the neuronal integrity, synaptic density, and the microglial and astrocytes expression in different brain regions. Increased microglial activation was found in all four subregions (Figure 5). GFAP expression was found to be significantly elevated in the CbCo and somatosensory cortex (Figure 7), reflecting the glial activation response in the infected aged, humanized mice. As a marker of axon-myelin stability, the decreased MAG expression in the CbCo, WB, and Hippo-CA1 regions (Figure 5) indicated the disruption of the axonal function due to HIV-associated aging and could be a cause of neuropathy. The decreased synaptophysin expression in the CbCo, WB, and Hippo-CA1 regions (Figure 6) suggested an overall reduction in synaptic density, a previously reported hallmark of cognitive impairment during the aging process [64].

We also looked at the expression of a new aging marker (EP2) in our brain samples. Prostaglandin E2 (PGE2) receptor 2 subtype (EP2) is a metabolite of arachidonic acid that binds and regulates PGE2-mediated cellular responses, and has been shown to be associated with several physiological and pathological events by acting through numerous signaling pathways in a wide range of tissues; it has also been implicated in many neurological diseases [65,66,67]. EP2 has been shown to play a role in aging [67], and its expression was found to be upregulated on virus-specific CTLs during chronic LCMV infection [68]. We observed an increased expression of the EP2 receptor in several brain regions (CbCo, WB, and Hippo-CA1) of the infected and aged hu-mice as compared to controls (Figure 6). More studies are needed on whether and how EP2 expression influences accelerated aging in PLWH. Additionally, neurofilament, a major neuron cytoskeletal component, was also found to be decreased in the CbCo, WB, and DG regions (Figure 7). In summary, glial activation, neuronal damage, and synaptic dysregulation were observed in infected aged hu-mice brains. However, the extent to which each marker was affected varied; for instance, astrocyte activation was observed in two cortical regions, whereas microglia activation was observed in both the cortical and hippocampal regions. Importantly, this observation was also supported by our transcriptomics analysis, suggesting that the NSG-humanized mice are a suitable model to study HIV-associated aging.

While the current study provided valuable insights between HIV infection and accelerated aging using the CD34-NSG hu-mice model, it has a few limitations. Though we observed prolonged viral presence and an increased activity of aging-related genes in our hu-mice, the interpretation of these findings to clinical settings should be made cautiously, keeping the complexities of human brain in mind. Our investigation focused on a specific subset of representative aging-related markers (84 genes) and its associated pathways during chronic HIV infection, and further research is needed to delve deeper into the cellular and molecular mechanisms of accelerated aging in PLWH. Future studies will aim to incorporate combinatorial ART treatment to comprehensively understand and mimic the viral suppression scenario related to aging in PLWH.

## 4. Materials and Methods

### 4.1. Generation and HIV-Infection of Hu-Mice

NSG (NOD.Cg-Prkdc^scid^ Il2rgt^m1Wjl^/SzJ) mice were generated and maintained under pathogen-free conditions at the University of Nebraska Medical Center (UNMC), following ethical guidelines for the care of laboratory animals at the National Institutes of Health. All experimental protocols were approved by the Institutional Animal Care and Use Committee (IACUC) at UNMC and as described [69]. To generate CD34^+^ humanized mice, NSG pups were irradiated with an RS 2000 biological irradiator (Rad Source Technologies Inc., Buford, GA, USA) after birth to eliminate rodent bone marrow stem cells, followed by the introduction of human CD34^+^ HSCs through intra-hepatic transplantation. The humanized mice (hu-mice) were generated in the current report by injecting CD34^+^ human HSCs into newborn immunodeficient NSG mice (50,000 cells/pup) as described previously [69]. To monitor human cell repopulation, peripheral blood was routinely collected starting from 12 weeks after HSCs transplantation and was analyzed using flow cytometry. These NSG mice were systematically reconstituted with human immune cells by 18–20 weeks in various tissue compartments, including brain sub-compartments such as the cortex, meninges, and brain stem, and have been shown to last up to 6–8 months [13,70]. At 20 weeks of age, humanized mice were selected based on the total human CD45+ cell count (>20%), and then divided into the uninfected control (n = 6) and HIV-1-infected group (n = 8). The HIV-1 infection of humanized mice was performed via an intraperitoneal injection of HIV-1_ADA_ at 1.5 × 10^4^ at 50% tissue culture infectious dose (TCID_50_) [71,72].

### 4.2. Flow Cytometry

Peripheral blood was collected into EDTA-coated tubes via submandibular bleeding at designated intermediate time points and via cardiocentesis at the study endpoint. After removing excess plasma, 50 μL of whole blood suspensions was incubated for 30 min with a combination of human monoclonal antibodies staining for CD45, CD3, CD19, CD4, CD8, and CD14, as described previously [71]. RBCs were lysed with FACS lysing solution (BD Biosciences, Franklin Lakes, NJ, USA) followed by fixation with 2% paraformaldehyde. Flow cytometry was carried out with a standard procedure using the Attune NxT Acoustic Focusing Cytometer system (Invitrogen, Thermo Fisher Scientific, Waltham, MA, USA). Human-cell-specific populations were analyzed using FlowJo v10.5 (BD Pharmingen, San Diego, CA, USA).

### 4.3. Viral Load Measurements

The levels of HIV-1 viral RNA were measured and analyzed at designated time points using the automated COBAS Ampliprep system v2.0/Taqman-48 system (Roche Molecular Diagnostics, Basel, Switzerland) as described previously [72], and plotted using Prism V9.4.1 software. The assay’s detection limit is 200 viral RNA copies/mL, reflective of the known dilution factors [71].

### 4.4. Immunohistochemical and Immunofluorescent Staining

Humanized mouse brains from control and HIV-infection aged groups were collected at the endpoint, fixed with 4% paraformaldehyde for 24 h, then processed using Epredia^TM^ STP 120 Spin Tissue Processor (Epredia^TM^, Thermo Fisher Scientific, Waltham, MA, USA) using the overnight protocol, and embedded in paraffin blocks. Five-micron-thick brain sections were acquired and stained with mouse monoclonal anti-human HIV-1p24 antibody, followed by counterstaining with Mayer’s hematoxylin for the nucleus. Images were captured under brightfield at 20 and 40× magnifications on the Nuance Multispectral Tissue Imaging system (CRi, Wobum, MA, USA).

For immunofluorescence staining, brain sections from the same paraffin blocks were blocked with 10% normal goat serum containing 0.5% tween in 1X tris buffered saline, followed by sequential 60 min incubation with primary antibodies to GFAP (Glial fibrillary acidic protein) (Dako), Iba1 (Ionized calcium-binding adaptor molecule (1) (Wako chemicals), NF (neurofilament) (clone2 F11; Dako), MAG (Myelin-associated glycoprotein) (sc-166780; Santacruz Biotech, Dallas, TX, USA), SYN (Synaptophysin) (clone SY38; EMD Millipore), and EP2 (Prostaglandin E2 receptor (2) (Abcam) alone or in combinations followed by 30 min incubation with secondary antibodies Fluor 488-conjugated goat anti-mouse IgG and/or Alexa-fluor 594-conjugated goat anti-rabbit (Invitrogen, Thermo Fisher Scientific, USA). DAPI (Invitrogen) was used for nucleus counterstaining. Images were taken in a fluorescence microscope at 40× magnification. For quantifications of different biomarkers, a minimum of 8 selected fields of view of the same brain region under 40× magnification was counted using Nuance Multispectral Imaging system.

### 4.5. Nucleic Acid Extractions and qPCR Assays

Total RNA from the brain was isolated from aged control and infected hu-mice using the RNeasy Mini Kit (QIAgen, Hilden, Germany) as per the manufacturer’s protocol. The RNA concentration was measured using a Nanodrop 2000 Spectrophotometer (Thermo Fisher Scientific, USA). RNA was then converted to cDNA with random hexamers using the cDNA synthesis kit (Invitrogen, Thermo Fisher Scientific, USA). HIV-1 gag and human CD45 gene were amplified using semi-nested real-time qPCR using a separate set of primers and probes as described previously [72,73]. The expressions of human CD45 and HIV-1 gag were analyzed using TaqMan gene expression assays. Tissue viral RNA and DNA loads were expressed as copies/million human-CD45-positive cells [14].

### 4.6. cDNA Synthesis and RT^2^ Profiler PCR Array Specific for Human Aging

A total of 500 ng RNA from one brain hemisphere from all the individual brain samples was used for cDNA synthesis using the RevertAid Frist Strand cDNA Synthesis Kit (Thermo Fisher Scientific, USA). RNA, nuclease-free water, Oligos, 5× reaction buffer, RiboLock RNase Inhibitor, dNTP Mix, and Revert Aid M-MulV Reverse Transcriptase were mixed to make the desired reaction volume. The cDNA synthesis reaction was performed for 60 min at 42 °C. The cDNA products were directly used for RT^2^ profiler PCR Array.

The RT^2^ profiler Human Aging 96-well PCR Array (QIAgen, catalog No: 330231) was employed to determine the genomic expressions of human aging associated with genomic instability, telomere attrition, neurodegeneration and synaptic transmission, epigenetic alterations, inflammation response, apoptosis, transcriptional regulation, and cellular senescence pathways. The array was designed to look at 84 human aging-associated genes. Five housekeeping genes, genomic DNA controls, and positive PCR controls were also incorporated into the array for normalization and negative/positive control purposes, respectively. The cDNA products from the previous step were amplified using RT^2^ SYBR^®^ Green qPCR Mastermix (QIAgen, catalog no. 330502), using the following PCR conditions (95 °C for 10 min followed by 40 cycles of 95 °C for 15 s and 60 °C for 1 min). The qPCR assay was performed on 17a Master cycler^®^ ep realplex system according to the manufacturer’s instructions (Eppendorf, Hamburg, Germany). Fluorescence intensity was measured and quantified to obtain the data sets.

### 4.7. Transcriptomics Analyses

The genomic expression profile of hu-mice brain tissues was standardized, and fold changes and statistical significance were determined using Qiagen’s RT^2^ Profiler analysis software (Qiagen, Hilden, Germany) (https://geneglobe.qiagen.com/us/analyze) from the aged control and HIV-infected group. Differentially (upregulated and/or downregulated) expressed genes were identified based on the statistical analysis using *p*-values < 0.05. IPA (Qiagen, Hilden, Germany) (https://www.qiagen-bioinformatics.com) was used to identify and predict the functional pathways and networks affected in chronically HIV-infected and aged hu-mice as compared to controls after 40 weeks post infection.

### 4.8. Statistical Evaluations

Statistical analysis was performed using GraphPad Prism 9.4.1 software (GraphPad Software, San Diego, CA, USA). The differences in the mean values between groups were analyzed using Student’s *t* test or one-way analysis of variance (ANOVA). *p* values of less than or equal to 0.05 were considered statistically significant. Transcriptomics analysis to find statistically significant fold changes in gene expression was performed using Qiagen’s RT^2^ Profiler analysis with the same *p*-value criteria as mentioned above. IPA was performed to identify enriched pathways with gene expression with absolute fold changes greater or equal to 1.5. CNS and immune-system-related pathways with a -log (*p*-value) cutoff of 1.3 were filtered and selected to generate the report.

## 5. Conclusions

With an ever-aging population of people living with HIV, understanding the interplay between HIV infection and aging is timely. However, the complex nature of aging, coupled with the chronic inflammation and comorbidities associated with HIV infection, necessitates a robust model system to study chronic HIV infection. Using our hu-mice model system in the current study, the following conclusions can be summarized. *First*, sustained viral presence for a longer period in our infected and aged NSG-hu-mice mirrors the chronic HIV disease progression. *Second*, the analyses of genes linked to human aging showed heightened activity in infected and aged mice as compared to their uninfected counterparts, validating accelerated aging for the first time in any in vivo long-term model of HIV infection, and revealed the specific genes and pathways affected due to chronic HIV infection. *Third*, immunofluorescence imaging and the quantification of aging-related neuronal and glial markers further validated accelerated aging in the brain. Using the CD34-NSG hu-mice model, we investigated the intersection between HIV infection and accelerated aging processes in the brain for the first time. Future studies to understand CNS biomarkers of immune activation, immune senescence, neuroinflammation, epigenetic alterations, and other cellular dysregulation processes in greater detail under ART treatment will allow a better diagnosis and the development of therapeutics for PLWH with age-related health challenges.

## Data Availability

All the data findings from this study are included within this manuscript, and any other relevant data supporting the key findings of this study are available from the corresponding authors upon reasonable request.
